# Supplementary material for: Pharmacological HDAC3 inhibition alters memory updating in young and old male mice
Source: Front Mol Neurosci. 2024 Jun 26;17:1429880. doi: 10.3389/fnmol.2024.1429880 (PMC11234845; doi:10.3389/fnmol.2024.1429880)
Supplement: Supplementary file 1 [file Data_Sheet_1.PDF]

Supplementary Material

FIGS1. POST-UPDATE RGFP966 IN AGED MICE

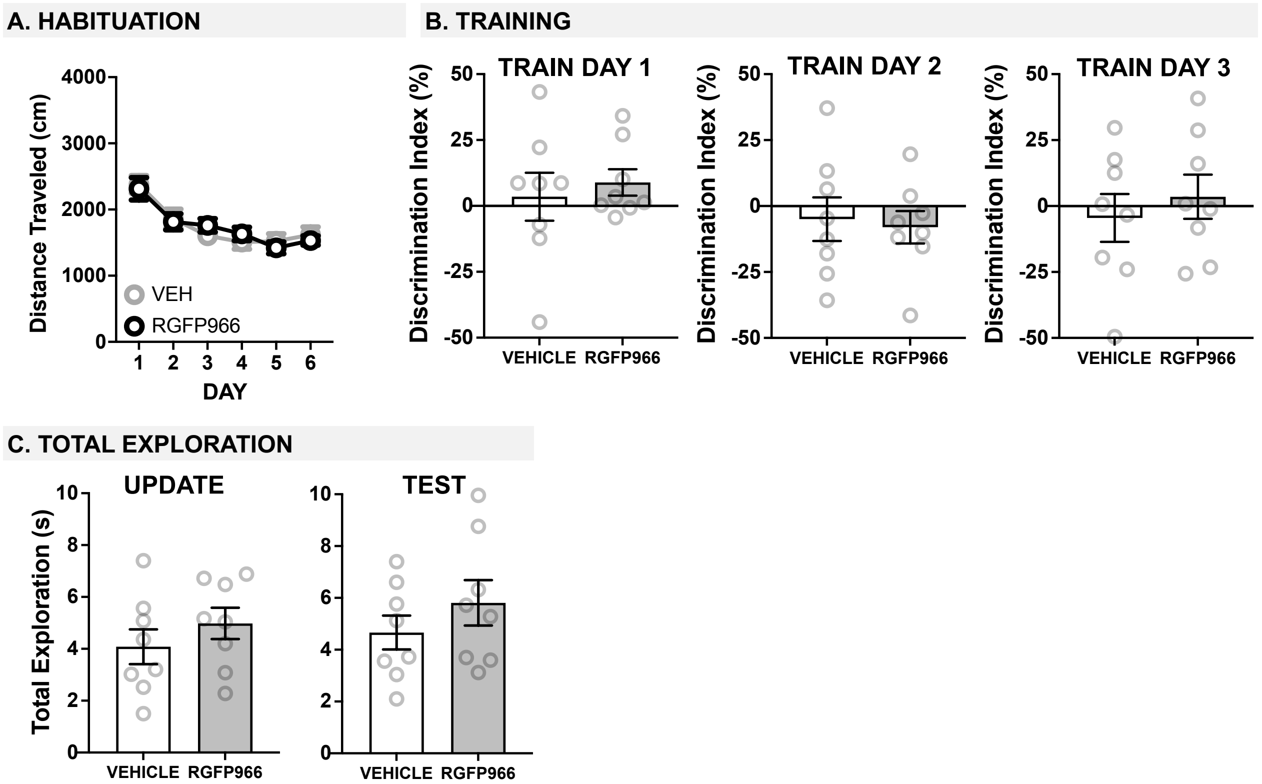

Supplementary Figure 1. *Post-update RGFP966 in aged mice* (**A**) Mice decreased their movement over six days during habituation, with similar distance traveled for vehicle and RGFP966 mice before injection. (**B**) Training DIs for each day of training. Vehicle and RGFP966 mice show similar, low DIs across each day. **(C)** Total exploration during the test session was similar for vehicle and RGFP966 mice. (*p<0.05, **p<0.01, ***p<0.001)

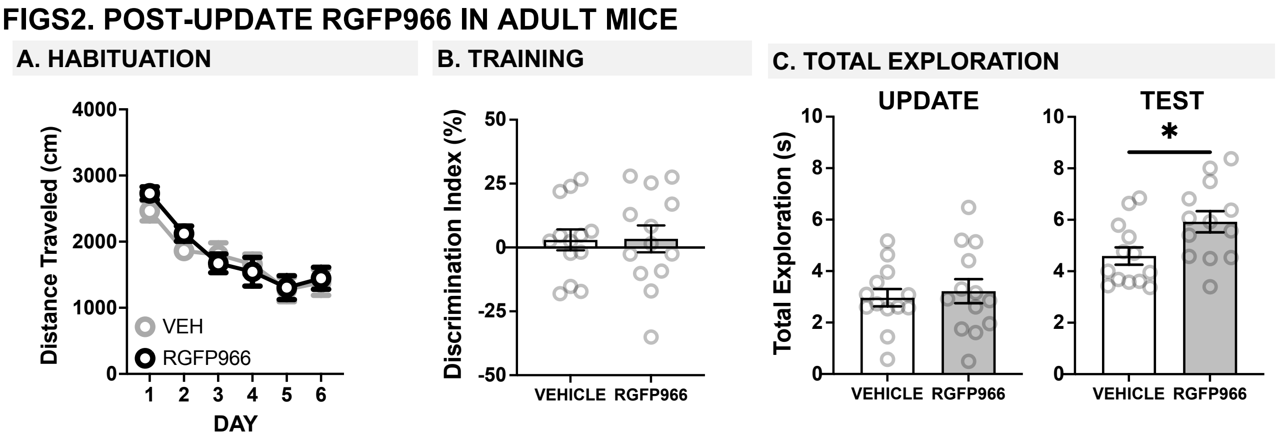

Supplementary Figure 2. *Post-update RGFP966 in young mice* (**A**) Mice decreased their movement over six days during habituation, with similar distance traveled for vehicle and RGFP966 mice before injection. (**B**) Training DIs for training. **(C)** Total exploration during the test session was higher for RGFP966 compared to vehicle mice. (*p<0.05, **p<0.01, ***p<0.001)

FIGS3. POST-SUBTHRESHOLD-UPDATE RGFP966 IN ADULT MICE

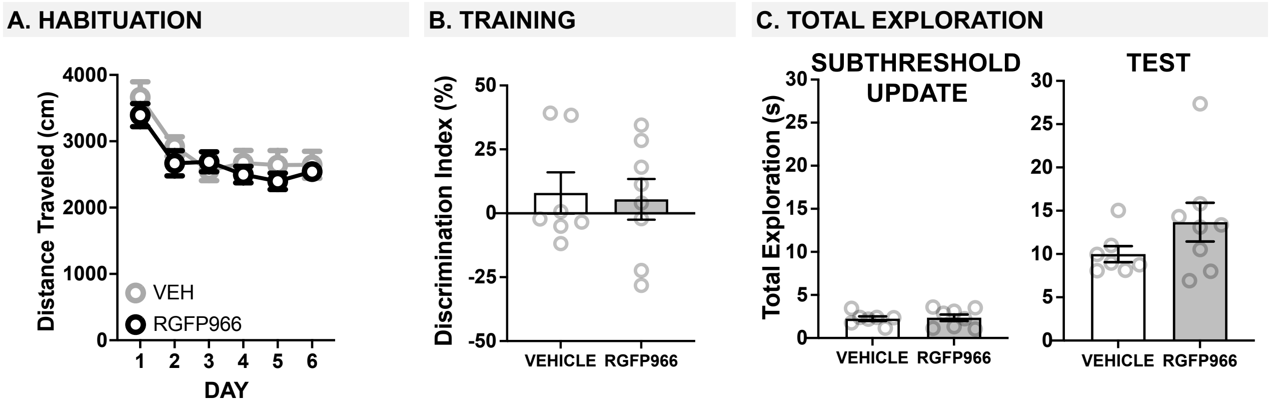

Supplementary Figure 3. *Post-subthreshold-update RGFP966 in adult mice* (**A**) Mice decreased their movement over six days of habituation, with similar distance traveled for vehicle and RGFP966 mice before injection. (**B**) Training DIs were similar for vehicle and RGFP966 mice before injection. **(C)** Total exploration during the test was similar for vehicle and RGFP966 mice. (*$p<0.05$, **$p<0.01$, ***$p<0.001$)
